# Liver tumor segmentation based on 3D convolutional neural network with dual scale

**DOI:** 10.1002/acm2.12784

**Published:** 2019-12-02

**Authors:** Lu Meng, Yaoyu Tian, Sihang Bu

**Affiliations:** ^1^ College of Information Science and Engineering Northeastern University ShenYang China

**Keywords:** 3D convolution, convolutional neural network, CT image of liver, multiscale, tumor segmentation

## Abstract

**Purpose:**

Liver is one of the organs with a high incidence of tumors in the human body. Malignant liver tumors seriously threaten human life and health. The difficulties of liver tumor segmentation from computed tomography (CT) image are: (a) The contrast between the liver tumors and healthy tissues in CT images is low and the boundary is blurred; (b) The image of liver tumor is complex and diversified in size, shape, and location.

**Methods:**

To solve the above problems, this paper focused on the human liver and liver tumor segmentation algorithm based on convolutional neural network (CNN), and specially designed a three‐dimensional dual path multiscale convolutional neural network (TDP‐CNN). To balance the performance of segmentation and requirement of computational resources, the dual path was used in the network, then the feature maps from both paths were fused at the end of the paths. To refine the segmentation results, we used conditional random fields (CRF) to eliminate the false segmentation points in the segmentation results to improve the accuracy.

**Results:**

In the experiment, we used the public dataset liver tumor segmentation (LiTS) to analyze the segmentation results qualitatively and quantitatively. Ground truth segmentation of liver and liver tumor was manually labeled by an experienced radiologist. Quantitative metrics were Dice, Hausdorff distance, and average distance. For the segmentation results of liver tumor, Dice was 0.689, Hausdorff distance was 7.69, and the average distance was 1.07; for the segmentation results of the liver, Dice was 0.965, Hausdorff distance was 29.162, and the average distance was 0.197. Compared with other liver and liver tumor segmentation algorithms in Medical Image Computing and Intervention (MICCAI) 2017 competition, our method of liver segmentation ranked first, and liver tumor segmentation ranked second.

**Conclusions:**

The experimental results showed that the proposed algorithm had good performance in both liver and liver tumor segmentation.

## 1 INTRODUCTION

Liver is one of the largest and most important organs in the human body, and the liver is also one of the organs with a high incidence of malignant tumors. Liver cancer is a major threat to human health, whose incidence is increasing every year worldwide.^1^ Accurate measurement of liver tumor size from abdominal computed tomography (CT) images, segmentation, and localization of tumor areas are helpful for clinicians to make an accurate evaluation of liver tumors. Currently, liver tumor segmentation is manually performed by radiologists on hundreds of CT images slice by slice, which is very tedious and time‐consuming, and the segmentation results depend on the clinical knowledge and experience of the radiologists. Therefore, automatic liver and liver tumor segmentation algorithm is essential and helpful for computer‐aided diagnosis. The main difficulties of automatic liver and liver tumor segmentation algorithm are: (a) liver is very close to adjacent organs and their CT values are similar to each other; (b) the CT contrast between the liver tumor and healthy tissue is low, and the boundaries around liver tumor are blurring; (c) the shape, size, and location of liver tumors are complex and variable.

The segmentation algorithms for liver and liver tumors were mainly divided into four categories: regional growth,^2^, ^3^ graph cut,^4^, ^5^, ^6^ level set,^7^, ^8^ and deep learning.^9^, ^10^, ^11^, ^12^, ^13^, ^14^, ^15^ The segmentation algorithm in this paper was based on deep learning, so we mainly reviewed several classic liver and liver tumor segmentation algorithms based on deep learning. Ben‐Cohen et al.^15^ used the VGG16 architecture of the fully convolutional network (FCN) for liver segmentation and liver lesion detection. They discarded the final classifier layer of VGG16 and converted all fully connected layers into convolutional layers. A two‐channel convolution was added to predict the probability of lesions or the liver at each output location, and then the output is up‐sampled to the original pixel for end‐to‐end learning using a deconvolution layer. Sun et al.^14^ designed a multichannel FCN to segment liver tumors from enhanced CT images. Because each stage of the enhanced CT data provided unique information about the pathological features, the method trained a network for each stage and then fused their high‐level features. In the research of Qi et al.,^10^ a three‐dimensional (3D) depth supervision network based on FCN was proposed. The network had a full convolution architecture, which was an end‐to‐end approach to learn and predict. The most important innovation structure of this network was the depth supervision of hidden layer, which can accelerate the speed of optimization convergence and improve the prediction accuracy. Finally, based on the high‐quality score map generated by the 3D depth monitoring network, the contours were refined using the fully connected conditional random field to obtain fine segmentation results. Based on FCN, Ronneberger et al.^12^ proposed a U‐Net network model. The entire neural network consisted of two main components, namely the contraction path and the extension path. The contraction path was mainly used to capture the context information in the picture, and the extension path was to accurately locate the part of the image that needed to be segmented. Ronneberger et al. also proposed the data enhancement method for training some data with small samples, especially the data related to medicine, and proved that U‐Net was very helpful for deep learning in medical images with small samples. Therefore, the structure of U‐Net was widely used in the research of medical image segmentation. Han et al.^13^ combined U‐Net's long‐distance cascade connection with ResNet's short‐range residual connection. The model had 32 layers, the input of the model was composed of several adjacent axial CT image slices, and the output was a two‐dimensional (2D) segmentation map corresponding to the input center slice. Patrick et al.^11^ proposed a segmentation algorithm using two cascaded U‐Net networks on CT slices. The first network was only used to segment the liver, and the mask map of the liver region generated by the first step was taken as the input of the second network to train the second U‐Net network, and the second network was only used to segment the tumor. Finally, the conditional random field was applied to the full dataset to obtain the relationship between the slices.

In MICCAI 2017 competition of liver and liver tumor, 20 liver segmentation algorithms and 24 liver tumor segmentation algorithms were proposed, and the ranking of these algorithms is shown in Tables 6 and 7. Almost all the ranking top algorithms used U‐Net or VGG‐Net, and the best one obtained 0.961 Dice for liver segmentation and 0.686 Dice for liver tumor segmentation. However, none of these algorithms directly used 3D CT images in the whole neural network, because training 3D image data in a complicated convolutional neural network was time‐consuming and required high computational resources.

To solve the above‐mentioned problems, this paper proposed a TDP‐CNN, which can fuse the local features with the global contextual information from the background, and directly processed 3D medical image data to obtain the 3D spatial information, and greatly speed up the training procedure. Furthermore, a conditional random field was combined in our algorithm to fine‐segment the results from TDP‐CNN.

## 2 MATERIALS AND METHODS

### 2.1 Algorithm overall flow

**Figure 1:**
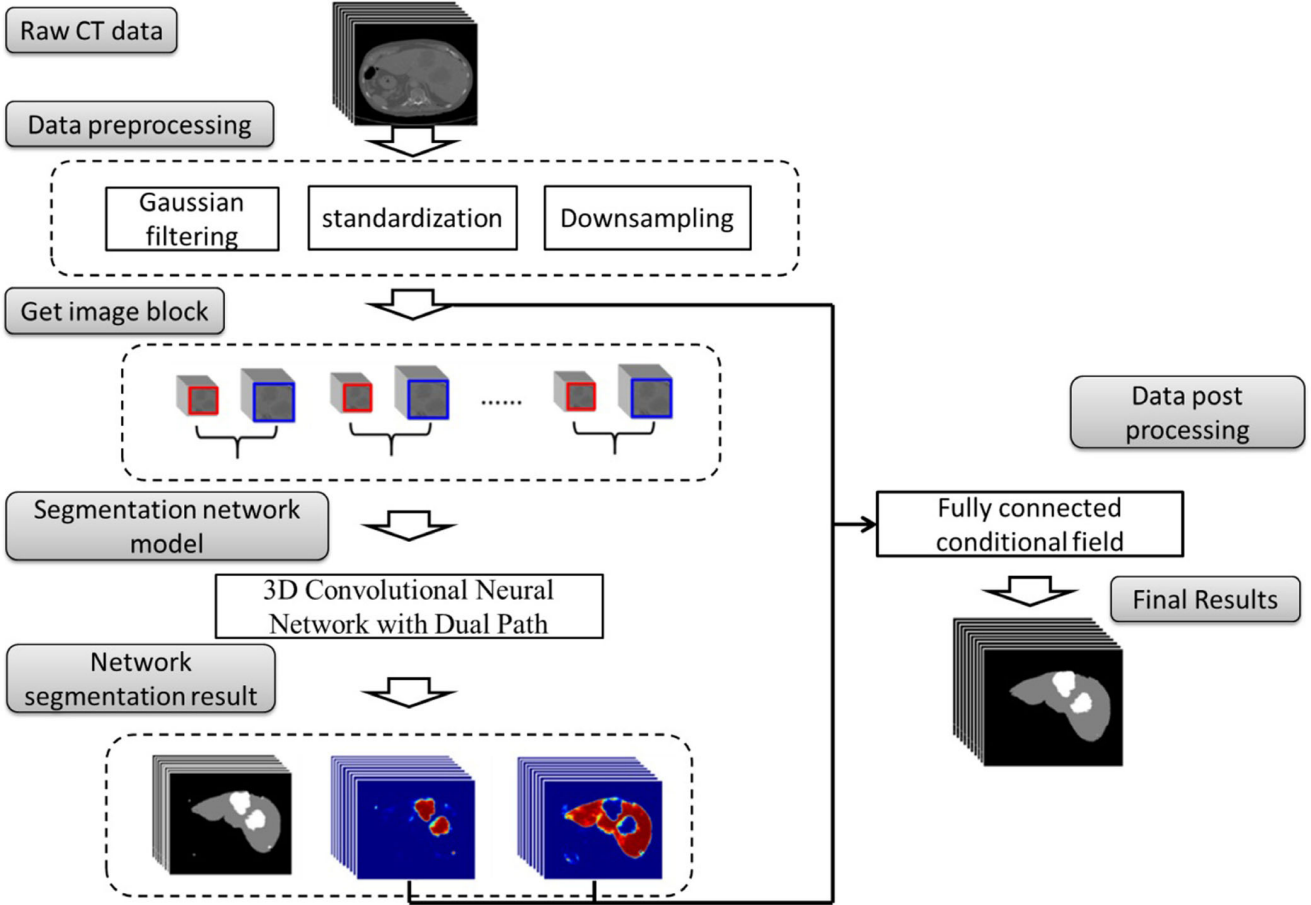
Flow chart of our method.

**Figure 2:**
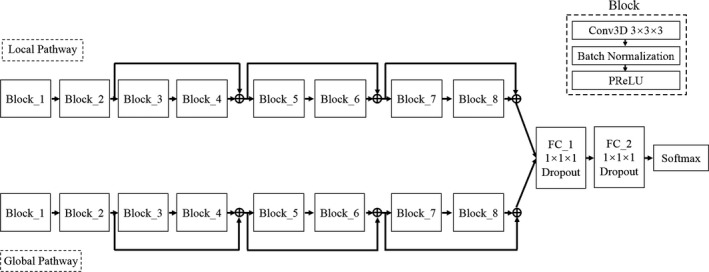
Schematic of three‐dimensional dual path‐convolutional neural network (TDP‐CNN) model, there were two paths in the model, one for local and the other for global, the architecture of both paths were totally the same. In each path, there were eight blocks, and all the blocks had the same architecture, which was composed of a 3 × 3 × 3 3D convolution layer, a batch normalization layer, and a PReLu layer. And residual connections were employed between block 2 and block 4, between block 4 and block 6, and between block 6 and block 8. And the end of two paths, the feature maps were fused and input into the fully connected layers and softmax layers to get the final classification results.

The algorithm proposed in this paper was mainly based on a 3D convolutional neural network with the dual scale from two paths. Shown in Fig. 1, the overall scheme of the algorithm was as follows:

1. Filtered and normalized the original CT images;
2. Segmented the 3D CT images into several sub‐image blocks, which were used as the input of TDP‐CNN. The architecture of TDP‐CNN was shown in Fig. 2. There were two paths in the TDP‐CNN, and each path was composed of eight blocks, and all the blocks had the same architecture, which included one convolutional layer, one batch normalization layer, and one activation layer. The feature maps of two paths were fused, and input into the fully connected layer, and then classified in the softmax layer.
3. The trained TDP‐CNN was used to segment the liver and liver tumor, and generate probability maps of the segmentation results;
4. Finally, the probability maps were post‐processed by a fully connected conditional random field algorithm to obtain the final segmentation results of liver and liver tumors.

### 2.2 Data preprocessing

Before liver tumor segmentation, we used Gaussian smoothing to filter the CT images to remove the noise caused by the equipment and environment.

(1) $$H_{i,j}=\frac{1}{2\pi \sigma^{2}}e^{-\frac{{\left(i-k-1\right)}^{2}+{\left(j-k-1\right)}^{2}}{2\sigma^{2}}}$$

where k denotes the dimensional filtering kernel width and σ denotes the standard deviation, and in this paper σ = 1. Then, the filtered CT images were further normalized; each pixel was normalized to the mean and standard deviation of the whole image, so that the pixel values of all CT images meet the standard normal distribution. Besides, due to the computation limits of our workstation, the CT images were subsampled from 512 × 512 to 256 × 256 to reduce the amount of computation. Finally, data augmentation was used to deal with the small dataset size, and we geometrically rotated, flipped, cropped, and scaled the original CT images so that we can obtain more variant liver and liver tumor types and enlarge our training dataset.

### 2.3 TDP‐CNN architecture

**Figure 3:**
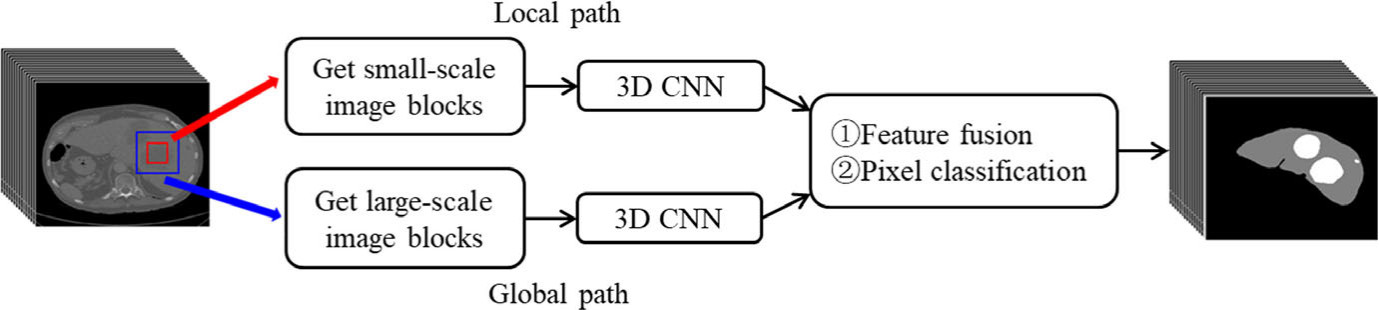
Simplified schematic three‐dimensional dual path‐convolutional neural network.

As it is known that there are limited computational resources in computer system, such as CPU power, GPU power, memory size, data transferring speed et al., and 3D medical image data take much more memory than 2D image data, and all the 3D convolutional network’s operations, such as convolution, pooling, activation et al. also take much more computational time than 2D operations. Therefore, complicated CNN, like U‐Net or VGG‐Net, always has a heavy computational burden and maybe trained for weeks or even months, if the 3D medical image data were directly loaded into CNN. But CNN with simple architecture cannott have good performance of liver and liver tumor segmentation results. To balance the computational performance and the requirements of computational resources, three improvements were made: (a) We did not load the whole 3D medical image data from one subject into our CNN in one time, instead, we segmented the whole 3D medical image data into small segments, and only several segments were input into our CNN each time; (b) To capture the 3D spatial features, we used multiscale small segments. Here, “multiscale” refers to two segments with the same center, but one segment has a bigger image size and higher image resolution, the other segment has a smaller image size and lower image resolution. (c) To compute the multiscale segments together in our CNN, we specially designed a dual path neural network architecture. Here, “dual path” refers to a local path and global path, respectively, shown in Fig. 3. In the local path, segments with smaller image sizes but higher image resolution were loaded, processed, and trained to capture the image features of local details, such as contour, texture, and so on. Similarly, in the global path, segments with bigger size but lower image resolution were loaded, processed, and trained to capture the image global features, such as background and contextual information. Then the features maps from two paths were fused at the end of the local and the global path, and transferred to the fully connected layer and a softmax layer.

According to the network architecture of our method, several important parameters had to be determined first, such as the kernel size of the 3D convolutional operations and the 3D size of the small segments. To determine the optimal combination of these parameters, we needed to repeat the experiments of selection many times. In this selection process, we only needed to know which combination was the best. And this best combination of parameters always remained the best one no matter in single‐path or two‐path convolutional neural networks. Therefore, we only needed a simple one‐path convolutional neural network to perform the experiments of parameter combination selection, and the simple network structure can save us a lot of time and resources. We referred AlexNet as this simple one‐path convolutional neural network, which contained five convolutional layers, three down‐sampling layers, and three fully connected layers. We did not use pooling layers, because the pooling operation will result in the loss of the exact location of the voxels, which may harm the accuracy of the segmentation results.

For a 3D convolutional neural network, the calculation of 3D convolutional operations costs much more computational resources than 2D convolutional operations. Therefore, only the kernel sizes of 3 × 3 × 3 and 5 × 5 × 5 were under consideration, but 5 × 5 × 5 kernel had about 4.6 times more parameters than 3 × 3 × 3 kernel. To build a deeper convolutional neural network, in this paper, we chose 3 × 3 × 3 as the kernel size of our convolutional operations.

The size of the 3D image segment was another important parameter, and we can obtain it based on the size of the receptive field.

(2) $$R_{l}^{\left\{x,y,z\right\}}=R_{l-1}^{\left\{x,y,z\right\}}+\left(k_{l}^{\left\{x,y,z\right\}}-1\right)\times \prod_{i=1}^{l-1}s_{i}^{\left\{x,y,z\right\}}$$

*R_l_*, *k_l_*, *s_i_* were 3D vectors of {x, y, z}, *R_l_* represented the size of the receptive field in the *l*‐th layer, *k_l_* represented the size of the convolution kernel, *s_i_* indicated the stride size of the *i‐*th layer, and the size of receptive field in the first layer was 1.

**Figure 4:**
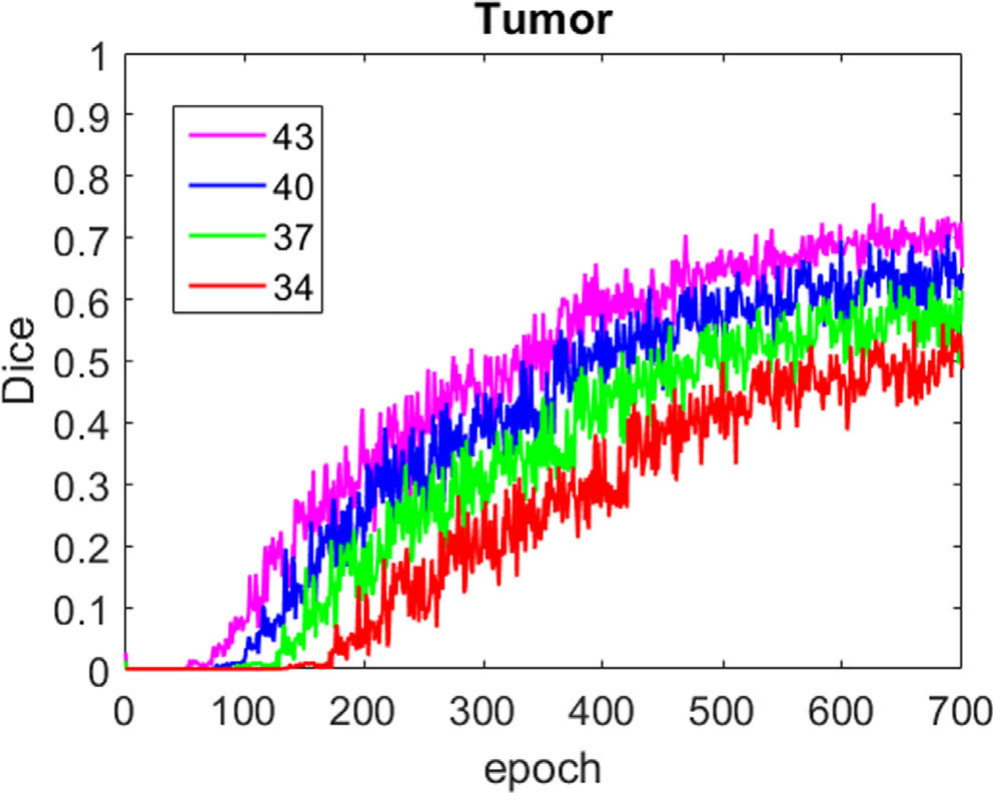
Influence of different three‐dimensional image block sizes on local path segmentation performance. (a) Comparison of dice and (b) comparison of sensitivity.

In our simple one‐path CNN, which was used to testify the combination of parameters, *l *= {1,2,…,7,8}, *i *= {1,2,…,*l*‐1}, *R_0_* = 1, *k_l_* = 3, *s_i_* = 1, according to formula (2), *R_8_* = 17, and the size of the receptive field of the last layer was 17 × 17 × 17. This result meant that one voxel in the last layer represented the features of 17 × 17 × 17 voxels in the original 3D image, which indicated that the minimal size of the 3D image segment was 17 × 17 × 17. We selected four different sizes to train the simple one‐path CNN, which were 34 × 34 × 34, 37 × 37 × 37, 40 × 40 × 40, and 43 × 43 × 43, and the segmentation results of these four trained model were compared based on Dice and sensitivity, shown in Fig. 4. We found that as the size of 3D image segments grew bigger, Dice and sensitivity also increased, which meant that 3D CNN could benefit from the bigger size of 3D image segments no matter one‐path or dual path architecture. Therefore, we selected 43 × 43 × 43 as the size of 3D image segments. More specifically, in the local path, we used 43 × 43 × 43 image segments as the input to the CNN (shown as the red rectangle in Fig. 3), and in the global path, we cut 129 × 129 × 129 image segment from original 3D image whose center was the same as the 43 × 43 × 43 segments (shown as the blue rectangle in Fig. 3), then subsampled it to 43 × 43 × 43, and input it to the CNN.

**Figure 5:**
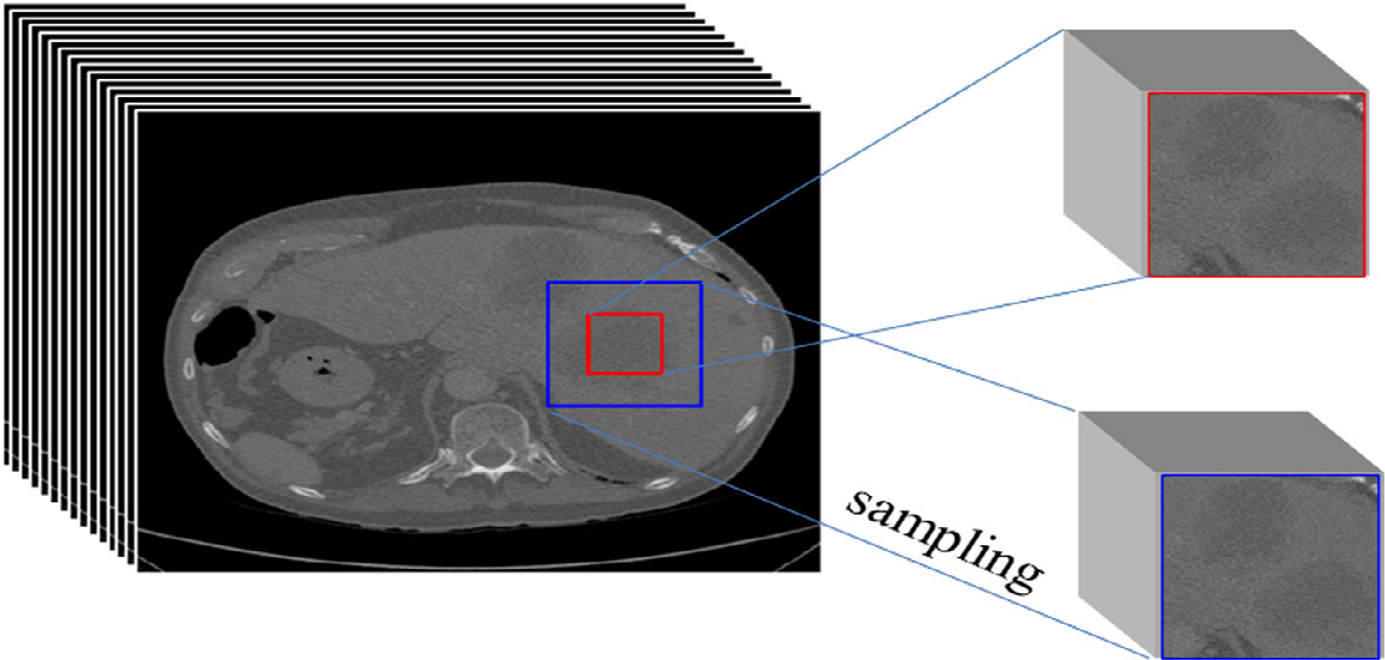
Sampling method of three‐dimensional image segment in the local path and global path; the red region represents the sample segment in the local path and the blue region represents the sample block in the global path.

We can learn more context information by processing large‐scale image segments from the global path. Due to the high computational cost of 3D networks, we subsampled the large‐scale image segments from 129 × 129 × 129 to 43 × 43 × 43, shown as Fig. 5. Although the input 3D image segments of the two paths had the same size, they contained different 3D image information and features, more details in the local path and more context information in the global path.

After we determined the size of 3D kernel and the size of 3D image segments, we needed to analyze the architecture of the 3D CNN, shown as Fig. 2. There were two paths in the TDP‐CNN, one was the local path, the other was the global path, and these two paths had the same architecture. There were eight blocks in each path, and every block was composed of one 3D convolutional layer with kernel size 3 × 3 × 3, one batch normalization layer, and one PReLu layer. Batch normalization was a technique for improving the performance and stability of 2D CNN, and can also be used in 3D CNN to normalize the input layer by adjusting and scaling the activations and mitigate the problem of internal covariate shift. In TDP‐CNN, we used residual connections between block 2 and block 4, block 4 and block 6, block 6 and block 8, to further illustrate the residual connections, we took the residual connection between blocks 2 and 4; for example, the outputs of block 2 were directly transferred to the end of block 4, and the outputs of blocks 2 and 4 were added together. The residual connections can give later layers direct access to feature maps of previous layers, which can improve gradient propagation resulting in faster convergence during training and better neural network performance. At the end of both paths, the outputs from the local path and global path were added together, to obtain the feature maps about the images' local details and global spatial information. Then these feature maps were transferred to 3D fully connected layers and considering that the number of feature maps was *Q*, the size of 3D feature maps was *M* × *N* × *P*. In regular CNN, the operations of fully connected layers consisted of two steps; firstly, the *Q* feature maps were convolved with a kernel, whose size was also *M* × *N* × *P*, in order to transform one feature map from 3D matrix to one element, so the number of parameters can be greatly reduced, but the spatial information was lost, and secondly, the *Q* elements were connected to every neuron in the fully connected layer. However, in our TDP‐CNN, the first step of fully connected layer operation was different, that is, the *Q* feature maps were convolved with a kernel, whose size was *1* × *1* × *1*, in order to keep the size of the feature maps unchanged, so our model had more parameters in this layer, but it can maintain the 3D spatial information, which was very important for 3D liver and liver tumor segmentation. Finally, the outputs of fully connected layers were input to softmax classifier to obtain the probability maps of liver and liver tumor segmentation results. Until now, the whole architecture of TDP‐CNN was depicted.

More specifically, the neural network parameters of each layer are shown in Table 2, and other parameters in the training of TDP‐CNN are shown in Table 3.

### 2.4 Data post‐processing

To remove the mis‐segmentation points, this paper used fully connected CRFs (FC‐CRF).^16^, ^17^ Considering that there were *N* pixels in a CT image, each pixel corresponded to a CT value set *I = *{*I_1_*,*I_2_*,…,*I_N_*} and a category label set *L*={*l_1_*,*l_2_*,…,*l_k_*}, *k* = 3 in this paper, because there were three categories (liver, liver tumor, and background), and the set of category labels was $X=\left\{X_{1},...,X_{N}\right\}$. Then the conditional random field (*I, X*) can be represented by the Gibbs distribution:

(3) $$P\left(\text{X|I}\right)=\frac{1}{Z(I)}\exp\left(-E\left(X|I\right)\right)\;$$

*Z(I)* was a constant, and E(*X |I*) represented the label distribution of the CT image and the Gibbs energy function when the pixel points were distributed as *X* and *I*. The conditional random field assigned a label to each pixel by solving the maximum posterior probability of the label $x\in L^{N}$:

(4) $$x^{*}=\arg\max_{x\in L^{N}}P\left(x|I\right)$$

Therefore, the process of solving conditional random fields (CRF) was the process of minimizing the Gibbs energy function. E(*X|I*) was defined as shown in Eq. (7), where *i* and *j* took values from 1 to *N*:

(5) $$E\left(\text{X|I}\right)=\sum_{i}\Psi_{\mu }\left(x_{i}\right)+\sum_{i<j}\Psi_{p}\left(x_{i},x_{j}\right)$$

$\Psi_{\mu }\left(x_{i}\right)$ was a one‐dimensional potential function, which was calculated by the classifier independently for each pixel, indicating that the pixel *i* was divided into the energy of the label $x_{i}$. In our method, the one‐potential function was calculated by the probability map of the liver and liver tumor generated by the TDP‐CNN model. The binary potential function $\Psi_{p}\left(x_{i},x_{j}\right)$ indicated that the pixels *i*, *j* were simultaneously divided into the energy of the label $x_{i}$ and $x_{j}$, and its expression was:

(6) $$\Psi_{p}\left(x_{i},x_{j}\right)=\mu \left(x_{i},x_{j}\right)\sum_{m=1}^{K}\omega^{\left(m\right)}k^{\left(m\right)}\left(f_{i},f_{j}\right)$$

$k^{\left(m\right)}$ was a Gaussian kernel:

(7) $$k^{\left(m\right)}\left(f_{i},f_{j}\right)=\exp\left(-\frac{1}{2}{\left(f_{i},f_{j}\right)}^{T}\Lambda^{\left(m\right)}\left(f_{i}-f_{j}\right)\right)$$

$f_{i}$ and $f_{j}$ were the eigenvectors of pixels *i* and *j* in the feature space, respectively, $\omega^{\left(m\right)}$ were linear combination weights, and *μ* was a label compatibility function that satisfied the Potts model. Each kernel function $k^{\left(m\right)}$ had a symmetric, positive definite precision matrix $\Lambda^{\left(m\right)}$. For multiclass image segmentation, the potential function was defined by the color *I_i_*, *I_j_* of the pixel *i*, *j* and the position *P_i_* and *P_j_* of pixel *i*, *j*:

(8) $$k\left(f_{i},f_{j}\right)=\omega^{\left(1\right)}\exp\left(-\frac{{\left|p_{i}-p_{j}\right|}^{2}}{2\theta_{\alpha }^{2}}-\frac{{\left|I_{i}-I_{j}\right|}^{2}}{2\theta_{\beta }^{2}}\right)+\omega^{\left(2\right)}\exp\left(-\frac{{\left|p_{i}-p_{j}\right|}^{2}}{2\theta_{\gamma }^{2}}\right)$$

Based on Eqs. (4), (5), and (8), our method used FC‐CRF to give the same category labels to the pixels with large similarities, and give different category label to the pixels with small similarities.

## 3 EXPERIMENT

### 3.1 Experimental dataset and experimental platform

All liver CT images used in this experiment were from liver tumor segmentation (LiTS), which were from the MICCAI 2017 competition. LiTS was obtained from six different clinical sites, with pixel distance from 0.55 to 1.0 mm and slice spacing from 0.45 to 6.0 mm. LiTS consisted of 131 enhanced CT image sequences. Each CT sequence covered the entire abdomen and part of the chest cavity, and the file format was Nifti. The number of axial slices was not fixed, which ranged from 74 to 987, and the resolution of each CT slice was 512 × 512. The dataset also provided the ground truth of liver and liver tumors segmentation results, which were manually labeled by medical clinicians. To train and test our method, we used 81 CT sequences as the training dataset, 25 CT sequences as the testing dataset, and 25 CT sequences as the verification dataset. In the LiTS dataset, the patients have different types of liver tumor diseases, such as hepatocellular carcinoma and metastasis from other organs (lung, breast, and so on). Hyper or hypo‐dense contrast is used to enhance the image contrast. The number of tumors varies between 0 and 75, and the size of tumors varies between 38 and 349 mm^3^, and the HU value differences between tumor and liver vary between 0 and 98, whose mean is 31.94 and the standard deviation is 20.^21^ Software and hardware configuration in our experiments are shown in Table 1.

**Table 1:** Experimental environment of our method.

Environment	Configuration
GPU	NVIDIA GeForce GTX 1080
Memory capacity	8 GB
CPU	Intel Core i5‐7500 @3.4 GHz
Memory Capacity	16 GB
Hard drive capacity	1 TB
Operating system	Ubuntu 16.04
Software tools	Python 2.7; tensorflow 1.8; matlab 2015b

### 3.2 Segmentation performance quantitative evaluation index

In medical image segmentation, true positive (TP) measures the proportion of actual positives that are correctly identified, true negative (TN) measures the proportion of actual negatives that are correctly identified, false positive (FP) is an error in which a test result improperly indicates presence of a condition, false negative (FN) is an error in which a test result improperly indicates no presence of a condition. And we can obtain more indicators using TP, TN, FP, FN^18^, ^19^, ^20^:

1. Dice

(9) $$\text{Dice}=\frac{2\text{TP}}{2\text{TP}+\text{FP}+\text{FN}}$$

Dice is a commonly used indicator for evaluating the results of medical image segmentation. Dice is 100% when the prediction result is completely consistent with the real result.

- Sensitivity

(10) $$\text{Sensitivit}y=\frac{\text{TP}}{\text{TP}+\text{FN}}$$

Sensitivity, also known as true‐positive rate or recall rate, is used to measure the ability of the algorithm to identify positive data.

- Specificity

(11) $$\text{Specificit}y=\frac{\text{TN}}{\text{TN}+\text{FP}}$$

Specificity is used to reflect the ability of the algorithm to identify negative data. Besides, the experiments in this paper also use two distances between pixels to evaluate the segmentation results.

- Hausdorff distance

Considering that all the voxels in ground truth images are represented by $s_{g}$, and all the voxels in the predicted images are represented by $s_{p}$, the Hausdorff distance can be given:

(12) $$\text{HD}\left(s_{g},s_{p}\right)=\max\left(h\left(s_{g},s_{p}\right),h\left(s_{p},s_{g}\right)\right)$$

In the formula, $h(s_{g},s_{p})$ is called the one‐way Hausdorff distance and is given by:

(13) $$h\left(s_{g},s_{p}\right)=\max_{a\in s_{g}}\;\min_{b\in s_{p}}∥a-b∥$$

where ||·|| denotes the Euclidean distance. The Hausdorff distance is sensitive to outliers and is used to find the largest distance between the ground image and the predicted image.

- Average distance

The average distance, also known as the average symmetric surface distance (ASSD), is given by:

(14) $$\text{ASSD}\left(s_{g},s_{p}\right)=\frac{d\left(s_{g},s_{p}\right)+d\left(s_{p},s_{g}\right)}{N_{1}+N_{2}}$$

where *N_1_* and *N_2_* represent the number of voxels in $s_{g}$ and $s_{p}$, respectively, $d(s_{g},s_{p})$ denotes the average shortest distance between voxels from $s_{g}$ to $s_{p}$, and $d(s_{g},s_{p})$ can be calculated by:

(15) $$d\left(s_{g},s_{p}\right)=\frac{1}{N}\sum_{a\in s_{g}}\min_{b\in s_{p}}∥a-b∥$$

ASSD is used to represent the overall difference between two sets. For a completely correct segmentation result, ASSD value is 0, which means that the predicted image completely coincides with the real image.

### 3.3 TDP‐CNN parameter settings

*conv* represented the convolutional layer, the *kernel* represented the size of convolution kernels in each layer, and the *FMs* represented the number of feature maps in each layer.

In this experiment, the local path and global path of TDP‐CNN had the same network architecture, as shown in Table 2. In the training of TDP‐CNN, the initializing setting and parameters are shown in Table 3.

**Table 2:** Network parameters of the local path and global path in three‐dimensional dual path‐convolutional neural network.

conv_1		conv_2		conv_3		conv_4	
Kernel	FMs	Kernel	FMs	Kernel	FMs	Kernel	FMs
3 × 3 × 3	30	3 × 3 × 3	30	3 × 3 × 3	40	3 × 3 × 3	40

conv_5		conv_6		conv_7		conv_8	
Kernel	FMs	Kernel	FMs	Kernel	FMs	Kernel	FMs
3 × 3 × 3	40	3 × 3 × 3	40	3 × 3 × 3	50	3 × 3 × 3	50

**Table 3:** Parameters during the training of three‐dimensional dual path‐convolutional neural network.

Parameter	Set value
Input image block size	43 × 43 × 43
Global path downsampling factor	3
Initial learning rate	0.001
RMS_decay	0.9
RMS_momentum	0.6
RMS_epsilon	0.0001
L1 Regularization coefficient	0.00001
L2 Regularization coefficient	0.0001
Dropout	0.5
Batch_size	10
Epoch	2000

### 3.4 TDP‐CNN training and testing results

**Figure 6:**
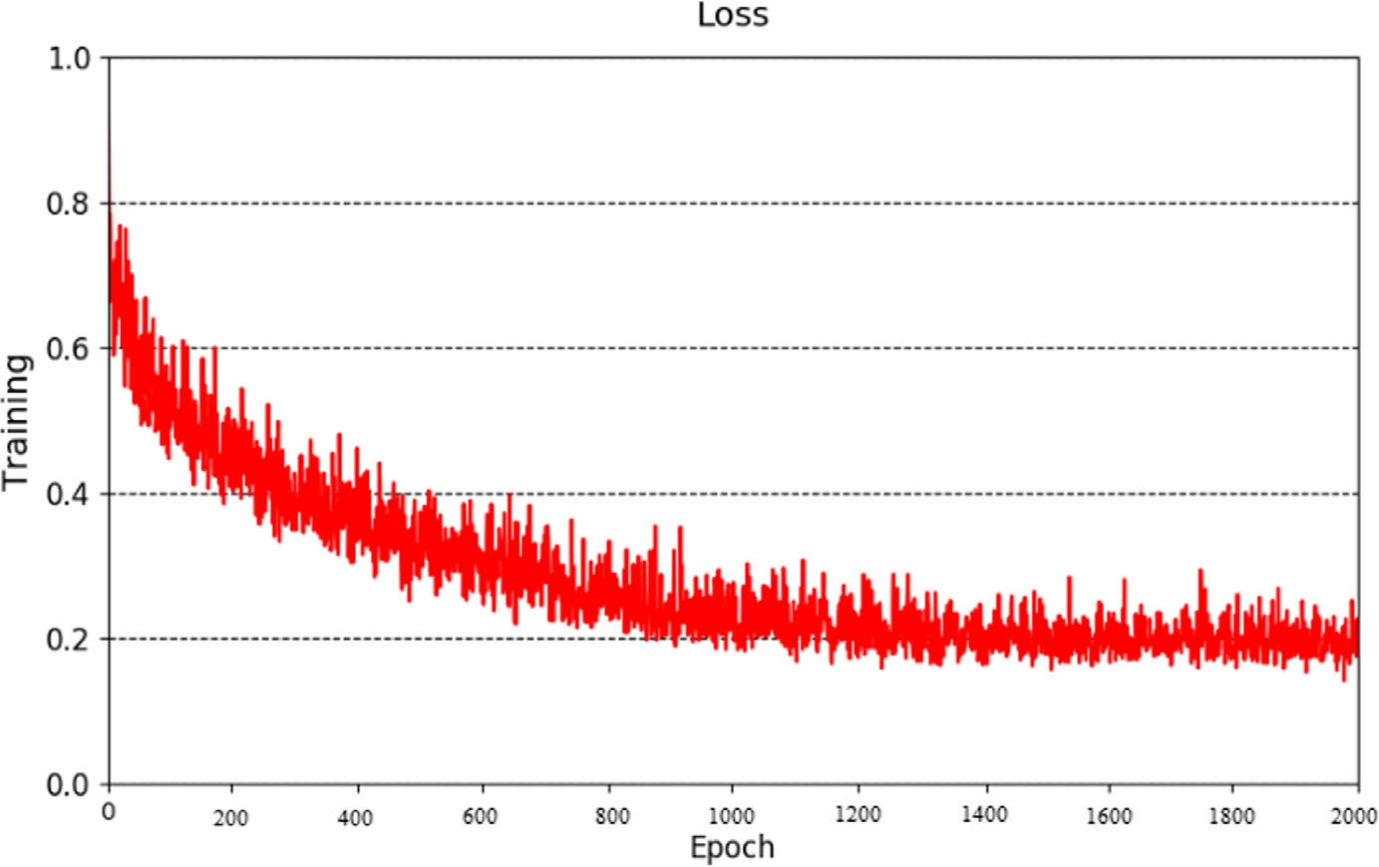
Loss function in the training.

During the training of TDP‐CNN, the ground truth of both liver and liver tumors was loaded into the network, so TDP‐CNN can simultaneously segment liver and liver tumors. The final classifier layer of the network divided the 3D CT images into three categories: liver, liver tumor, and background. In the experiment, we used the cross‐entropy as a loss function and used the random gradient descent algorithm to optimize the model. The loss function changed with the number of epochs, shown in Fig. 6, which was stable after 2000 epochs. And we had to point out that it took about 96 h to train the TDP‐CNN model for 2000 epochs.

During the testing, we evaluated three quantitative metrics, including Dice, Hausdorff distance, and an average distance of the algorithm's segmentation results on 25 samples from the test dataset. Table 4 shows the mean value of quantitative metrics of liver and liver tumor segmentation results about the 25 samples.

**Table 4:** Quantitative metrics of liver and liver tumors segmentation results.

	Dice	Hausdorff distance	Average distance
Liver segmentation	0.946	89.257	0.81
Tumor segmentation	0.688	65.384	4.005

**Figure 7:**
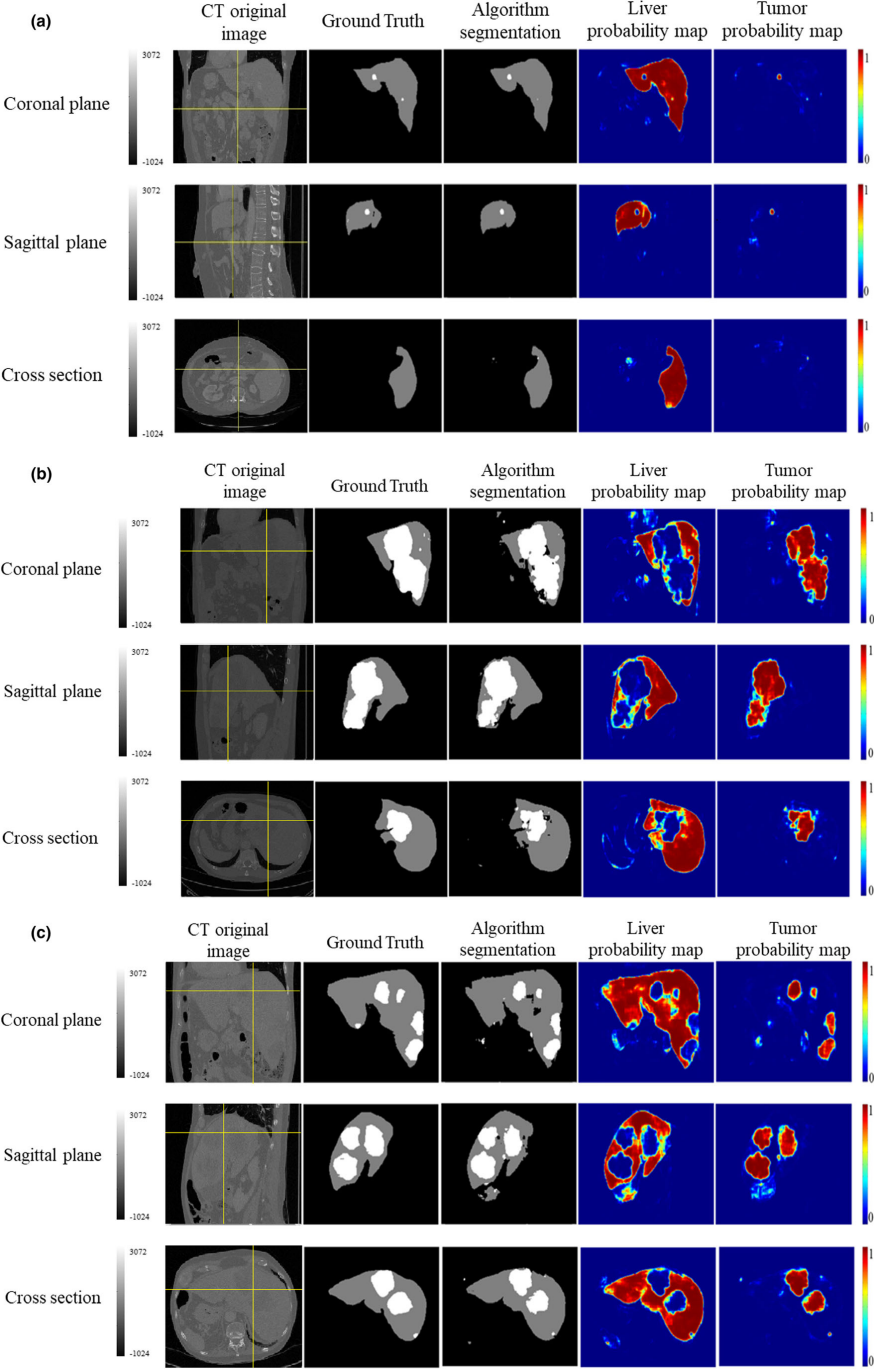
Ternary and probabilistic images of three‐dimensional dual path‐convolutional neural network liver and liver tumor segmentation results, crosshair indicated the 3D voxel in the liver. (a) Sample with small liver tumor, (b) Sample with a relatively large and connected liver tumor, and (c) Sample with a medium and unconnected liver tumor.

**Figure 8:**
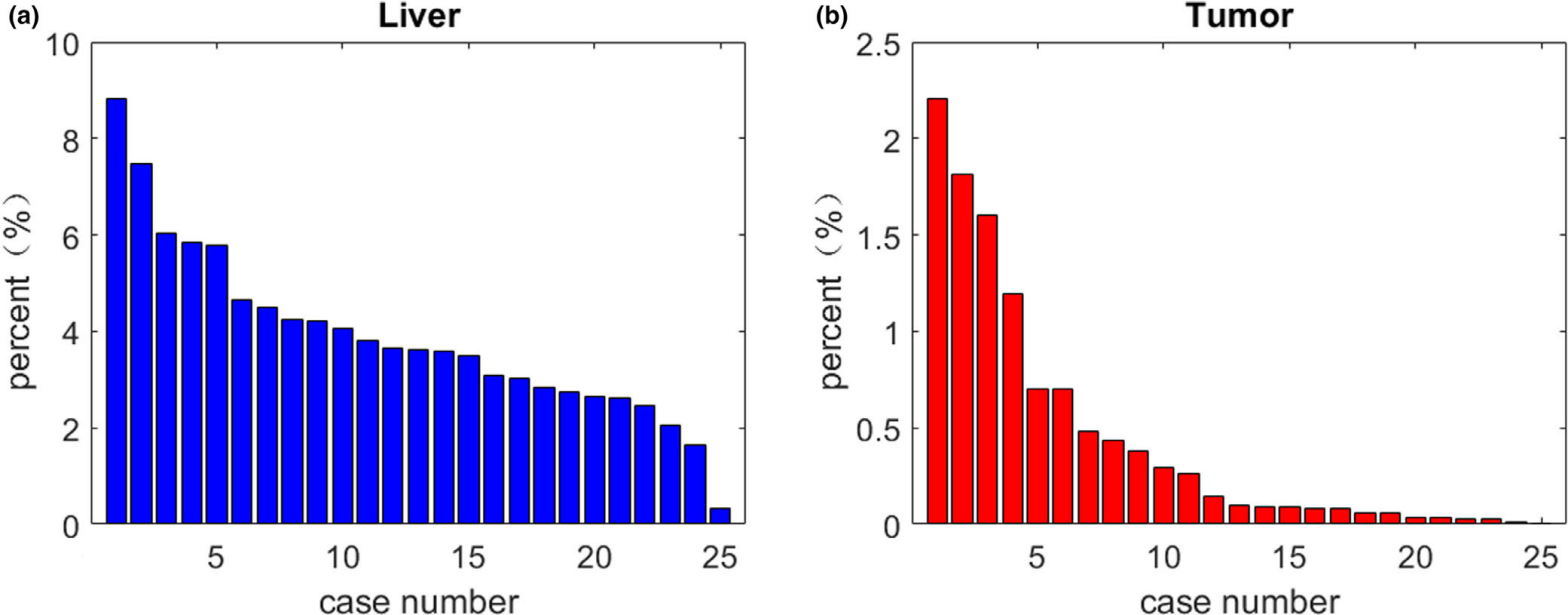
For all the 25 test samples, the percentage of liver voxels and liver tumor voxels in the whole computed tomography sequence, (a) Percentage of liver voxel and (b) Percentage of tumor voxel.

Liver and liver tumor segmentation results ares shown in Fig7. Based on the size of liver tumor, it can be divided into three types: (a) the size of liver tumors were small; (b) the liver tumors were relatively large and connected; (c) the liver tumors medium and not connected. As shown in Fig. 8, the size of liver tumors was extremely different from each other. In Fig. 7, from the left column to the right column, there were CT original images, the ground truth, the TDP‐CNN segmentation result, the liver probability map, and the liver tumor probability map, respectively. Comparing the TDP‐CNN segmentation results with ground truth, we can see that our method can successfully detect small liver tumor regions, except the ones near the lower part of the liver. It can be observed from Figs. 7(b) and 7(c) that our method performed well in the segmentation of relatively large liver tumor no matter the tumor was connected or unconnected.

However, we found that Hausdorff distances for liver and liver tumor segmentation were large, which meant that there were some false‐segmentation voxels. Therefore, we used FC‐CRF to refine the segmentation results to improve the segmentation accuracy. Table 5 shows the mean value of quantitative metrics of liver and liver tumor segmentation results about the 25 samples after using FC‐CRF. Comparing with Table 4, the Hausdorff distance of the liver segmentation result was reduced from 89.257 to 29.162, and the Hausdorff distance of the liver tumor segmentation result was reduced from 65.384 to 7.69.

**Table 5:** After optimizing the segmentation result of three‐dimensional dual path‐convolutional neural network using the fully connected conditional random field, quantitative indicators statistics of segmentation results of liver and liver tumors.

	Dice	Hausdorff distance	Average distance
Liver segmentation	0.965	29.162	0.197
Tumor segmentation	0.689	7.69	1.982

**Figure 9:**
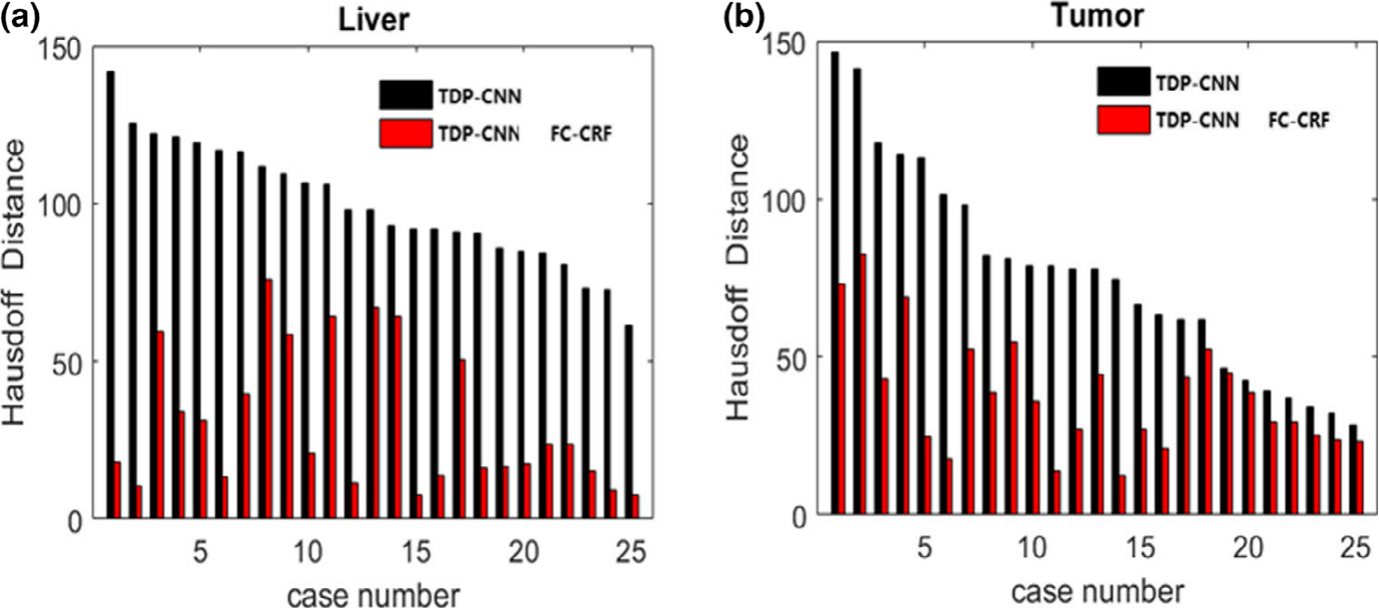
After optimizing the segmentation result of three‐dimensional dual path‐convolutional neural network (TDP‐CNN) by the FC‐CRF, the Hausdorff distance of liver and liver tumors is greatly reduced. Black bars mean using only TDP‐CNN to get the segmentation result, red bars mean using TDP‐CNN and fully connected‐conditional random fields to get the segmentation result. (a) Comparison of Hausdorff Distance for liver segmentation ad (b) Comparison of Hausdorff Distance for liver tumor segmentation.

**Figure 10:**
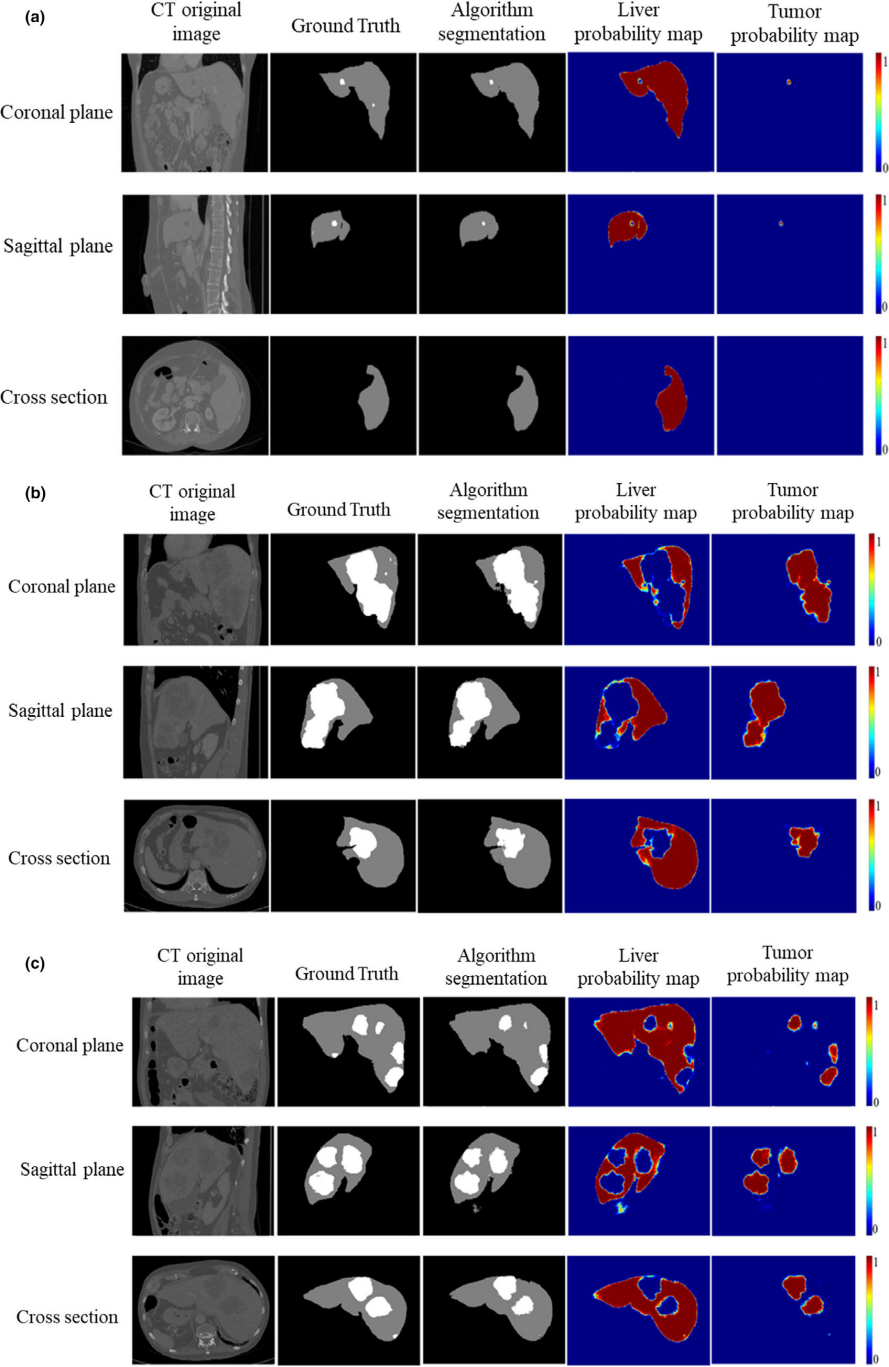
Segmentation results of liver and liver tumors using three‐dimensional dual path‐convolutional neural network (TDP‐CNN) and fully connected‐conditional random fields. (a) Sample with a small region of tumor, (b) Sample with a big region of tumor, and (c) Sample with multiple regions of tumor.

To more clearly show the optimization effect of FC‐CRF on TDP‐CNN, we compared Hausdorff distance for 25 samples in the test dataset. As shown in Fig. 9, the black blocks represented the Hausdorff distance of the liver and liver tumor segmentation result using only TDP‐CNN, and the red blocks represented the Hausdorff distance of the liver and liver tumor segmentation result using TDP‐CNN and FC‐CRF. We can conclude that the segmentation results of the liver and liver tumors were improved by FC‐CRF. The liver and liver tumor segmentation results using TDP‐CNN and FC‐CRF are shown in Fig. 10, and the image data were the same as the one in Fig. 7. It can be seen that the application of FC‐CRF can effectively remove false segmentation of liver and liver tumor. Finally, we had to point out that the average time taken by our method for each 3D CT image data was 13.3 min.

### 3.5 Comparison between our method and others

Finally, we compared our method with all the submitted methods of MICCAI 2017, including 20 liver segmentation methods and 24 liver tumor segmentation methods, all these methods used the same liver CT image dataset, which was LiTS, and the comparison results are shown in Tables 6 and 7. Limited by the length of this paper, we can only discuss the top three algorithms reported in the LiTS benchmark,^21^ whose authors are Li et al., respectively, and more details about other algorithms can be obtained from Ref. [21]. Li’s algorithm, ranked first, received 0.686 Dice value for liver tumor segmentation and 0.961 Dice value for liver segmentation. Li used cascaded U‐Net architecture to segment the liver and liver tumor, and weighted cross‐entropy was used as loss function during the training; furthermore, Li used residual connections by implementing a so‐called DenseNet architecture consisting of dense blocks in which a feature map takes the outputs of all previous feature maps as an input via a residual connection giving another boost to the information flow through the network and mitigating the vanishing gradient problem. Chlebus's algorithm, ranked second, received 0.676 Dice value for liver tumor segmentation and 0.96 Dice value for liver segmentation. Chlebus firstly used a small 3D network as the final network in a cascaded infrastructure to fuse the segmentation results of various previous networks into a final segmentation mask, then he trained three different U‐Net derived networks on resampled axial, sagittal, and coronal slices, respectively, for liver segmentation and combines them via the aforementioned small 3D network. This allows each network to view tumors and learn distinguishing features along a different coordinate axis and then combine its knowledge with the other networks to yield a more holistic multi‐axes aware classifier. Yuan’s algorithm, ranked third, received 0.657 Dice value for liver tumor segmentation and 0.963 Dice value for liver segmentation. Yuan firstly augmented the dataset by elastic deformation, and also used cascaded U‐Net architecture with stacks of image slices along the z‐axis as input, which was called 2.5D architecture in the report, and Jaccard was used as loss function during the training.

**Table 6:** Comparison of liver segmentation between our method and others.

Group	Dice	Hausdorff distance	ASSD
Our method	0.965	29.162	0.197
Yuan et al.	0.963	23.847	1.104
Li et al.	0.961	29.411	1.692
Chlebus et al.	0.96	24.499	1.15
Ben‐Cohen et	0.96	24.45	1.13
LP777	0.96	31.225	1.51
Wu et al.	0.959	28.229	1.311
jrwin	0.958	27.732	1.36
Wang et al.	0.958	32.933	1.367
MICDIIR	0.956	35.653	1.847
Vorontsov et al.	0.951	29.769	1.785
Kaluva et al.	0.95	32.71	1.88
Roth et al.	0.95	31.93	1.89
huni1115	0.946	31.84	1.869
Han	0.94	51.22	2.89
Lipkova et al.	0.94	186.25	3.54
mbb	0.938	90.245	2.9
Bi et al.	0.934	321.71	258.598
Micro	0.932	33.588	2.182
MIP HQU	0.93	29.27	2.16
szm0219	0.93	61.894	3.974
jinqi	0.924	123.332	5.104
mahendrakhened	0.912	45.928	6.465
jkan	0.906	63.232	3.367
Piraud et al.	0.767	326.334	37.45
QiaoTian	0.05	90.47	31.56
Ma et al.	0.041	8240.644	8231.318

**Table 7:** Comparison of liver tumor segmentation between our method and others.

Group	Dice	Hausdorff distance	ASSD
Our method	0.689	7.69	1.07
Li et al.	0.686	6.055	1.073
Chlebus et al.	0.676	7.322	1.143
Vorontsov et al.	0.661	6.317	1.075
Yuan et al.	0.657	6.269	1.151
Ma et al.	0.655	9.363	1.607
Bi et al.	0.645	6.472	1.006
Kaluva et al.	0.64	7.25	1.04
Han	0.63	7.21	1.05
Wang et al.	0.625	6.983	1.26
Wu et al.	0.624	7.783	1.232
Ben‐Cohen et al.	0.62	8.06	1.29
LP777	0.62	6.716	1.388
Micro	0.613	10.087	1.759
Njrwin	0.613	7.649	1.164
mbb	0.586	8.079	1.649
szm0219	0.585	7.408	1.222
MICDIIR	0.582	7.723	1.588
Roth et al.	0.57	6.81	0.95
jkan	0.567	7.23	1.159
huni1115	0.496	9.03	1.342
mahendrakhened	0.492	7.515	1.441
Lipkova et al.	0.48	8.64	1.33
jinqi	0.471	14.588	2.465
MIP HQU	0.47	7.84	1.09
Piraud et al.	0.445	8.391	1.464
QiaoTian	0.25	11.72	1.62

Compared with other liver and liver tumor segmentation algorithms, the Dice of our method ranked first in terms of liver and liver tumor segmentation; however, the Hausdorff distance of the segmentation results of our method was larger, indicating that there were some mis‐segmentation voxels, which needed to be improved.

## 4 DISCUSSION

In this paper, we used TDP‐CNN to automatically segment liver and liver tumor from 3D abdominal CT images. Compared with previous liver and liver tumor segmentation algorithms, the biggest differences of our method were that TDP‐CNN had dual path and each path focused on individual image size and resolution and captured global and local features, respectively.

**Figure 11:**
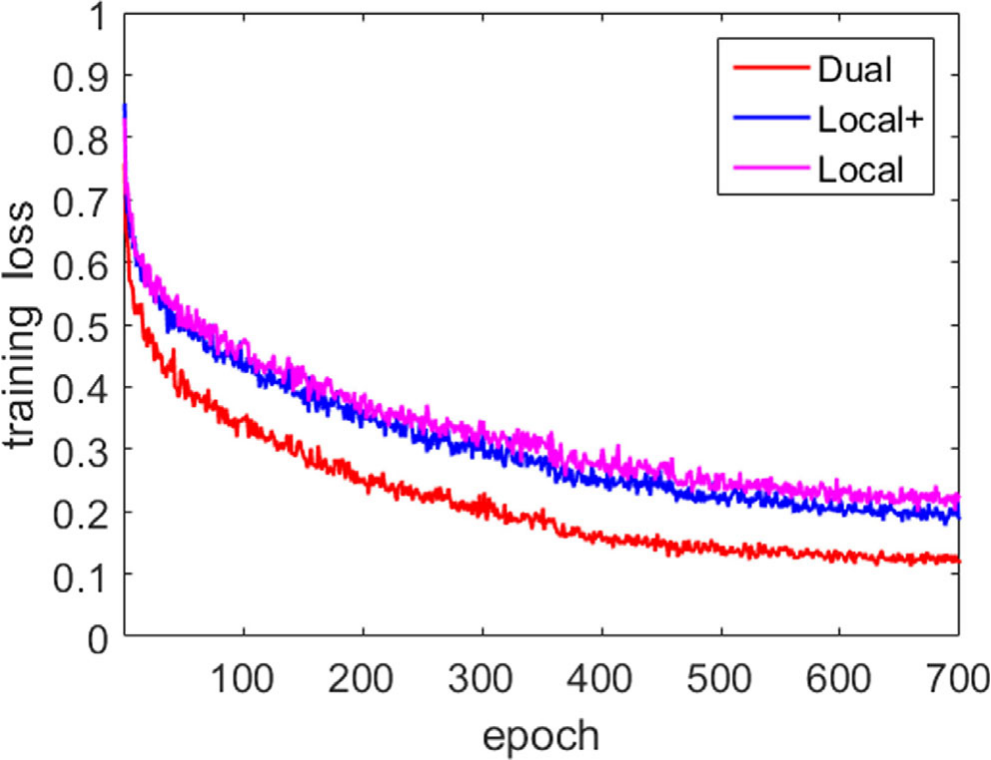
The comparison of segmentation performance between our dual path convolutional neural network (CNN) two sing‐path CNN, which are Local and Local+.

It is essential to compare the performance of dual path multiscale CNN with that of single‐path CNN, to clearly show the advantages of our method over the single‐path CNN. In the process of comparison, we built a single‐path CNN, whose architecture was the same as one of the two paths from TDP‐CNN, as shown in Table 2, and we named this single‐path CNN as “Local.” It can be clearly seen that the number of parameters from TDP‐CNN is two times more than that of Local, therefore, to make the comparison fair, we built another single‐path CNN, which also had eight layers and the same kernel size as shown in Table 2, but the number of feature maps in each layer was twice larger than that of Local, and we named this single‐path CNN as “Local+.” We trained all the three CNN 700 epochs, and the training loss is shown in Fig. 11, which indicated that our dual path multiscale CNN outperformed the other two single‐path CNN. Although the number of parameters from Local+ was two times more than that of Local, Local+ did not show an obvious advantage over Local, which means that the enhancement of the segmentation performance of our method benefits from the network architecture, not from the number of parameters.

Looking through all the algorithms reported in the MICCAI 2017 LiTS benchmark,^21^ most of the liver and liver tumor segmentation algorithms are based on deep learning, which benefits from vigorous development of deep learning on medical image processing and computer‐aided diagnosis in recent years. And U‐Net and VGG‐Net are the most used network structures. However, no algorithms specially designed a complete 3D convolution neural network or directly dealt with 3D medical image data, because the 3D operations of convolution and pooling are time‐consuming on training and require high resources from the computer station. Instead, some algorithms used a small 3D network to optimize the results from the previous 2D network or 2.5D network architecture to capture more characteristics from liver and liver tumor. And these algorithms, which partially used the 3D network, performed the top three in the liver and liver tumor competition; this result may indicate the feasibility and usefulness of 3D network architecture. In our method, we further utilized the 3D convolution neural network, and specially designed a complete 3D network architecture. To balance the segmentation performance and computational resource requirements, we used the dual path multiscale 3D network architecture, in which one‐path captured liver and liver tumor global features from bigger scale and smaller resolution and the other path captured the local features from smaller scale and bigger resolution, 3D medical image data were directly input into the network for training and testing. Our network can extract features from 3D images to the greatest extent, and reduce the computational resource requirements as much as possible.

Both our method and other algorithms from MICCAI 2017 competition had better liver segmentation results than liver tumor segmentation, which may be caused that human liver always has a relatively fixed shape but liver tumor's size and shape are diversified in size and type. Therefore, it may be more effective than if we specially design the liver tumor segmentation algorithm based on the size and type of the tumor.

Our method can accurately segment large liver tumor, but performed comparatively worse for small liver tumors, which is defined as a 3D size less than 500 voxels. The diameter of small liver tumor is only a few voxels, and the size of the whole liver image is 512 × 512; therefore, segmenting small structures in such a big background is a very difficult task. Besides, image noise and artifacts are also another influence on the task. This problem was also recognized by the algorithms in MICCAI 2017 challenge; some corresponding methods were developed, but still cannot get high Dice value. Therefore, more studies are needed in the future to improve the small liver tumor segmentation.

## 5 CONCLUSION

This paper proposed a TDP‐CNN architecture based on deep learning, which can be used to segment liver and liver tumor from the 3D abdominal CT images. The special design for 3D medical image data can make TDP‐CNN balance the segmentation performance and the requirement of computational resources. Compared with other liver and liver tumor segmentation algorithms, our method directly used 3D image data in the whole TDP‐CNN architecture, instead of 2.5D image data or small 3D network. Experiments showed that our method had Dice value 0.965 for liver segmentation and Dice value 0.689 for liver tumor segmentation. These quantitative metrics indicate that our method can accurately segment liver and liver tumor from 3D abdominal CT images.

## CONFLICT OF INTERESTS

The authors declared that they have no conflict of interest to this work. We declare that we do not have any commercial or associative interest that represents a conflict of interest in connection with the work submitted.

## References

[1] Chen Guoyong , Zhang Jiabin , Sun Jianjun , et al. Revisiting partial hepatectomy of large hepatocellular carcinoma in older patients. Sci Rep. 2018;8:14505. 30266965 10.1038/s41598-018-32798-0 PMC6162215

[2] Baâzaoui A , Barhoumi W , Ahmed A , et al. Semi‐automated segmentation of single and multiple tumors in liver CT images using entropy‐based fuzzy region growing. IRBM. 2017;38:98–100.

[3] Fekry Abd‐Elaziz O , Sharaf Sayed M , Ibrahim Abdullah M . Liver Tumors Segmentation from Abdominal CT Images using Region Growing and Morphological Processing. International Conference on Engineering and Technology; 2014:19–20.

[4] Beichel R , Bornik A , Bauer C , et al. Liver segmentation in contrast enhanced CT data using graph cuts and interactive 3D segmentation refinement methods. Med Phys. 2012;39:1361–1373. 22380370 10.1118/1.3682171 PMC4109564

[5] Chen X , Udupa JK , Bagci U , et al. Medical image segmentation by combining graph cuts and oriented active appearance models. IEEE Trans Image Process. 2012;21:2035–2046. 22311862 10.1109/TIP.2012.2186306 PMC5548181

[6] Boykov YY , Jolly M‐P . Interactive graph cuts for optimal boundary & region segmentation of objects in N‐D images. 8th IEEE Int Conf Comput Vis (ICCV); 2001;105–112.

[7] Li BN , Chui CK , Chang S , et al. A new unified level set method for semi‐automatic liver tumor segmentation on contrastenhanced CT images. Expert Syst Appl. 2012;39:9661–9668.

[8] Yinchun Z . Level set image segmentation based on rough set and new energy formula. Acta Automatica Sinica. 2015;41:1913–1925.

[9] Li X , Chen H , Qi X , et al. H‐DenseUNet: hybrid densely connected UNet for liver and tumor segmentation from CT volumes. IEEE Trans Med Imaging. 2017;99:1–13. 10.1109/TMI.2018.2845918 29994201

[10] Dou Q , Chen H , Jin Y , et al. 3D deeply supervised network for automatic liver segmentation from CT volumes. Med Image Comput Comput‐Assist Intervent; 2016:149–157.

[11] Christa PF , Ettlingera F , Gruna F , et al. Automatic liver and tumor segmentation of CT and MRI volumes using cascaded fully convolutional neural Networks. Medical Image Computing and Computer‐Assisted Intervention; 2016:415–423.

[12] Ronneberger Olaf , PhilippFischer Thomas Brox . U‐Net: Convolutional Networks for Biomedical Image Segmentation. Medical Image Computing and Computer‐Assisted Intervention; 2015:234–241.

[13] Han X .Automatic Liver Lesion Segmentation Using a Deep Convolutional Neural Network Method, arXiv preprint arXiv:170407239; 2017.

[14] Sun Changjian , ShuxuGuo HuimaoZhang , et al. Automatic segmentation of liver tumors from multiphasecontrast‐enhanced CT images based on FCNs. Artif Intell Med. 2017;83:58–66. 28347562 10.1016/j.artmed.2017.03.008

[15] Ben‐Cohen A , Diamant I , Klang E , et al. Fully convolutional network for liver segmentation and lesions detection. Deep Learning and Data Labeling for Medical Applications; 2016;77–85.

[16] Krahenbuhl P , Koltun V . Efficient inference in fully connected crfs with gaussian edge potentials. Conference and Workshop on Neural Information Processing Systems;2011:109–117.

[17] Zhuqiang L , Ruifei Z , Fang G . Hyperspectral remote sensing image classification based on three‐dimensional convolution neural network combined with conditional random field optimization. Acta Optica Sinica. 2018;38:404–413.

[18] Heimann T , van Ginneken B , Styner MA , et al. Comparison and evaluation of methods for liver segmentation from CT datasets. IEEE Trans Med Imaging. 2009;28:1251–1265. 19211338 10.1109/TMI.2009.2013851

[19] Chang H‐H , Zhuang AH , Valentino DJ , et al. Performance measure characterization for evaluating neuroimage segmentation algorithms. NeuroImage. 2009;47:122–135. 19345740 10.1016/j.neuroimage.2009.03.068

[20] Taha AA , Hanbury A . Metrics for evaluating 3D medical image segmentation: analysis, selection, and tool. BMC Med Imaging. 2015;15:29–57. 26263899 10.1186/s12880-015-0068-x PMC4533825

[21] Bilic P , Christ PF , Vorontsov E , Chlebbus G , et al.The Liver Tumor Segmentation Benchmark (LiTS), publish online: https://arxiv.org/abs/1901.04056

